# Time Spent for Activation of Non-Profit Studies in Oncology in Italy

**DOI:** 10.1371/journal.pone.0011864

**Published:** 2010-07-29

**Authors:** Gianfranco De Feo, Simona Signoriello, Jane C. Bryce, Antonia Del Giudice, Giuliana Canzanella, Federika Crudele, Fiorella Romano, Giovanni de Matteis, Manuela Florio, Fabiano Falasconi, Alfonso Savio, Pasqualina Giordano, Gennaro Daniele, Mario Iaccarino, Maria Carmela Piccirillo, Massimo Di Maio, Alessandro Morabito, Ciro Gallo, Francesco Perrone

**Affiliations:** 1 National Cancer Institute, Naples, Italy; 2 Department of Medicine and Public Health, Second University of Naples, Naples, Italy; University of Texas M. D. Anderson Cancer Center, United States of America

## Abstract

**Aim:**

The aim of this paper is to describe the time spent to activate oncological non-profit clinical trials promoted in Italy by the National Cancer Institute of Naples, following the implementation of recent European laws.

**Methodology:**

Data about the process of activation of 5 non-profit multicentre clinical trials were prospectively collected through a web-based system. The impact of European guidelines was assessed by comparing the efficiency of the process between applications started before and after the decree introducing in Italy the Clinical Trial Application form (MD-CTA). Outcomes of the descriptive analyses were the time to EC opinion, the time to administrative agreement signature after a positive EC opinion, and the cumulative percentage of submissions that came to closure (either positive or negative) within four subsequent time cohorts.

**Principal Findings:**

From March 2007 to October 2009, 202 applications were submitted to 107 centres. Forty-four (59%) applications of those submitted before were successful, compared to 71 (55%) of those submitted after MD-CTA. Most of the failures were due to missing EC response (27% and 22%) or administrative reasons (10% and 16%, before and after, respectively); very few (4% and 7%) were due to EC refusal. The impact of the MD-CTA on time to EC opinion looked positive (median 4.1 vs 2.4 months, before and after, respectively) but a subgroup analysis revealed that the impact was limited to a comparison biased by the selection of EC. After a positive EC opinion, there was no difference before and after MD-CTA in the time to administrative agreement signature (median 3.6 and 3.8 months, respectively). A trend to shortening time to closure of the whole submission process over the time was evident, with 58% of the applications coming to closure within 6 months from submission in the most recent cohort.

**Conclusions:**

In our experience there is reassuring evidence of a trend toward shortening the time spent to activate non-profit clinical trials in Italy, but the whole process still remains inefficient. Efforts should be made to improve the process, also focusing on administrative procedures.

## Introduction

**Figure 1:**
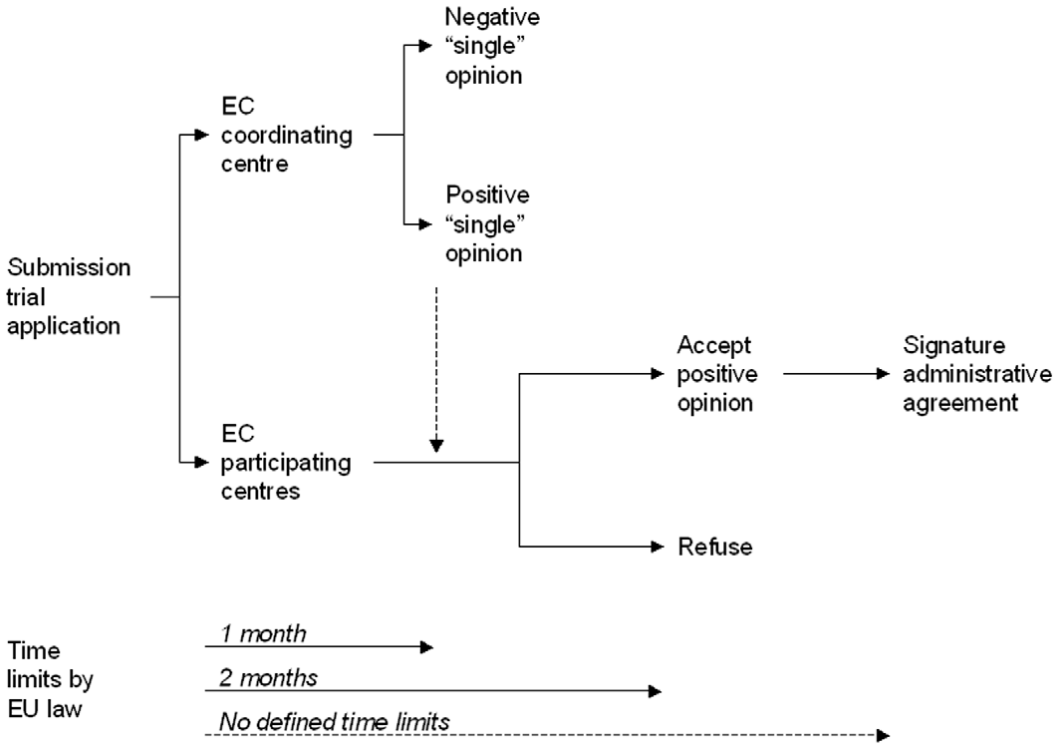
Flow of clinical trials application according to 2001/20/EC Directive.

Clinical research is regulated in Europe by the 2001/20/EC Directive, which should harmonize national laws and administrative provisions regarding clinical trials conduction [1]. In particular, the directive establishes at 60 days the maximum time to obtain the opinion of ethical committees (EC), including 30 days for issuing the so-called “single” opinion by the EC of the coordinating centre and 30 days for the acceptance or refusal of the “single” opinion by the EC of clinical centres willing to participate. The directive does not make any substantial difference between commercial (also defined “profit”) and academic independent (“non-profit”) clinical trials. In Italy, the European directive has been implemented with the Legislative Decree 211/2003; thereafter, further guidance has been provided with the implementation of Ministerial Decree 17 December 2004, that innovatively defined rules for non-profit clinical research [2], [3]. According to the European directive, the procedure for the activation of multicentre clinical trials foresees subsequent phases: first, the submission of the clinical trial application to EC of the coordinating and the participating centres; second, the issue of the “single” opinion by the EC of the coordinating centre and, if this opinion is positive, the acceptance or the refusal by EC of each participating centre; third, in case of acceptance, the signature of administrative agreement between the coordinating and each participating centre (**figure 1**).

The 7^th^ Bulletin of the National Monitoring Centre for Clinical Trials, reporting data on clinical trials of drugs in Italy in the years 2004–2007, showed that only 27.3% of peripheral EC issued their opinion within the time frame established by law, and that mean time of issuing EC opinion was 4.3 months [4]. A survey on the work of 134 Italian EC on a single trial reported a large variability of the procedures for clinical trial application, both for the number and the format of the documents. In fact, the number of required documents ranged from 6 to 21; at least one personalized document was required in most cases (57%) and the number of hardcopies ranged from 6 to 249 (5). Furthermore, 26.9% of EC asked for submission by e-mail or CD-ROM (number of copies ranging from 1 to 15) in addition to the paper version. In that survey, the median time from submission to final opinion was 72 days, longer than that mandated by the European Directive [5].

The scenario could have changed in Europe thanks to the publication of the detailed guidance on the modalities of clinical trial authorisation request to competent authorities [6], on the format of the application and the documentation to be submitted for EC opinion [7], and on investigational medicinal products and other medicinal products [8]. These guidelines were received in Italy in December 2007, with the Ministerial Decree on clinical trial application (MD-CTA) entering into force on July 4^th^, 2008 [9]. The MD-CTA defines all the operative procedures and the documents required for the authorisation of a clinical trial; it also establishes the maximum number of paper copies to submit to EC and introduces the CTA form, with the aim of uniquely identifying the clinical trial, the organisations and the key individuals responsible for the conduct of the trial.

The aim of the present study is to assess the impact of the MD-CTA on time to EC opinion and to administrative signature, and to describe the efficiency over time of the whole process of activation of non-profit multicentre clinical trials promoted by the Clinical Trials Unit of the NCI of Naples.

## Methods

Data on the process of activation of non-profit multicentre clinical trials coordinated by the Clinical Trials Unit of the NCI of Naples were prospectively collected through a web-based system (http://www.usc-intnapoli.net). Three time milestones were considered for each application: the date of submission to EC, the date of EC opinion, and the date of signature of administrative agreement.

The impact of MD-CTA was assessed by comparing the efficiency of the process between applications started before (i.e. between March 1^st^, 2007 and July 3^rd^, 2008) and after (i.e. between July 4^th^, 2008 and October 31^st^, 2009) it entered into force in Italy. Data for the analysis were frozen at January 31^st^, 2010. Time to EC opinion was calculated as the interval from submission to the date of EC opinion either negative or positive, censoring at January 31^st^, 2010, cases with no EC opinion. Time to administrative agreement signature after a positive EC opinion was calculated as the interval from the date of EC opinion to the date of signature, censoring at January 31^st^, 2010, cases with no signature. Both of these outcomes were described with Kaplan-Meier curves. The efficiency of the whole process of activation, affected by both time to EC opinion and time to administrative signature, was described for four consecutive cohorts of submissions, based on 8-month periods of time. A submission was considered closed either in case of signature of the administrative agreement or in case of negative EC opinion.

Due to the descriptive nature of the study, no formal statistical analysis was performed.

## Results

From March 1^st^, 2007 to October 31^st^, 2009, the Clinical Trials Unit of NCI of Naples promoted five multicentre clinical trials (**Table 1**). A total of 202 applications were submitted to the EC of 107 Italian centres, with a median of 2 (range 1–5) applications for each centre. In particular, 74 applications were made before and 128 after MD-CTA. Twenty-two EC received applications both before and after MD-CTA; 85 EC were involved only before (34) or only after (51) MD-CTA. Thirty-nine (36%) centres were located in northern Italy, 22 (21%) in central Italy and 46 (43%) in southern Italy or islands. In all cases, the application was prompted by the Investigator's declaration of the will to participate in the proposed clinical trials.

**Table 1:** Characteristics of the trials.

				Application to EC	
Trial	EudraCT number	Type of Cancer	Planned patients	Before MD-CTA entered into force (No. 74)	After MD-CTA entered into force (No. 128)
TORCH	2005-005968-90	NSCLC*	900	13	1
CAPPA – 2	2005-005631-97	NSCLC*	360	23	16
STAD – 1	2006-003995-36	SCLC**	160	38	4
MITO – 7	2008-001754-40	Ovarian	400	-	53
MITO – 8	2008-001755-22	Ovarian	250	-	54

*NSCLC: Non-small-cell-lung-cancer.

**SCLC: Small-cell-lung-cancer.

**Figure 2:**
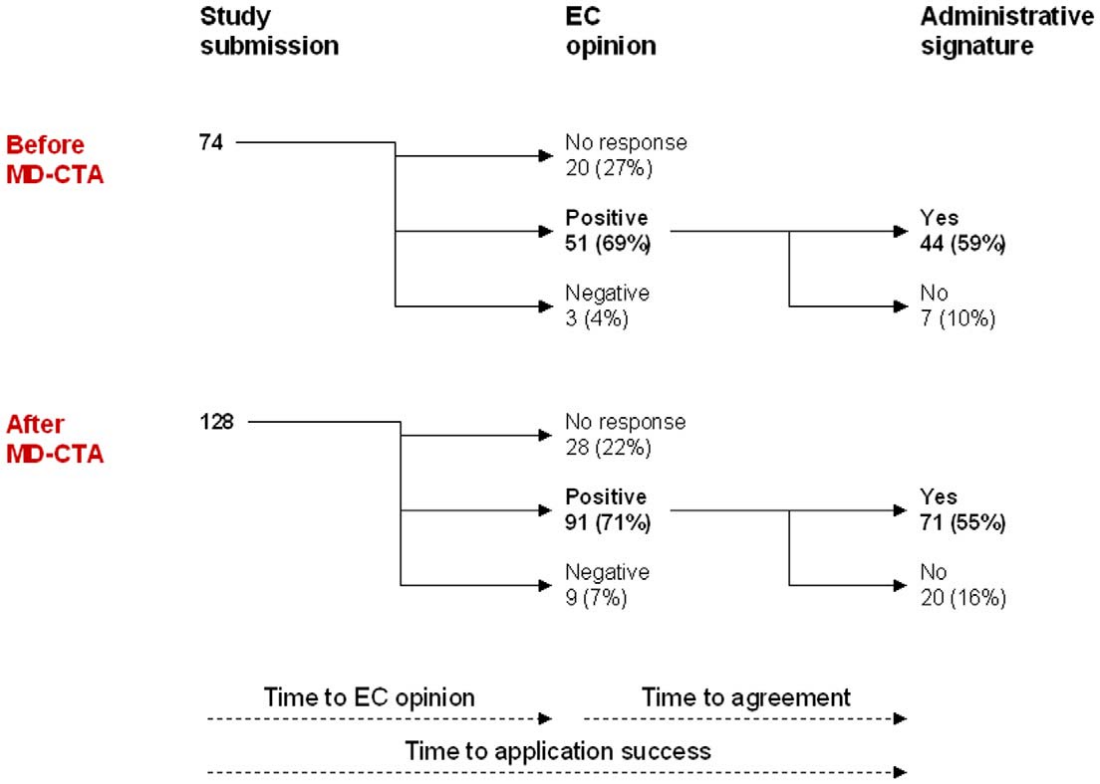
Data flow of application outcome before and after MD-CTA entered into force in Italy.

Forty-four (59%) applications submitted before MD-CTA were successful, compared to 71 (55%) of those submitted after. Failures in this process were similarly distributed before and after MD-CTA. Most of the failures were due to missing EC response (27% and 22% before and after, respectively) or administrative reasons following a positive EC opinion (10% and 16%, respectively); very few failures (4% and 7%, respectively) were due to EC negative opinion (**figure 2**).

**Figure 3:**
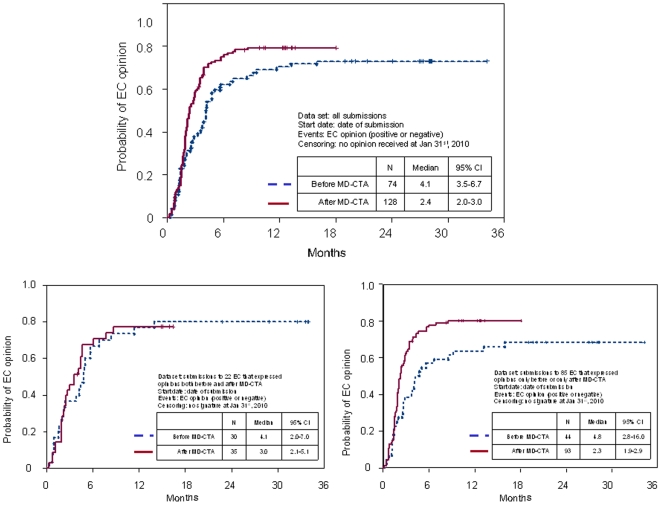
Time to EC opinion. Top graph includes all submissions; bottom left graph includes only data of EC involved both before and after MD-CTA; bottom right graph includes only data of EC involved only before or only after MD-CTA. Vertical lines represent censored applications.

Overall, median time to EC opinion was 2.9 months and only 37% of the opinions were issued within the 2-month time window indicated by law. The impact of MD-CTA on time to EC opinion is displayed in **figure 3**; the top graph, including all submissions, suggests that time to EC opinion was shorter after (median 2.4 months) than before (median 4.1 months) MD-CTA. However, bottom graphs show that there was no difference in time to EC opinion if the analysis is limited to EC that were involved both before and after MD-CTA (on different clinical trials), while a large difference was evident in the comparison including EC that were involved only before or only after MD-CTA.

**Figure 4:**
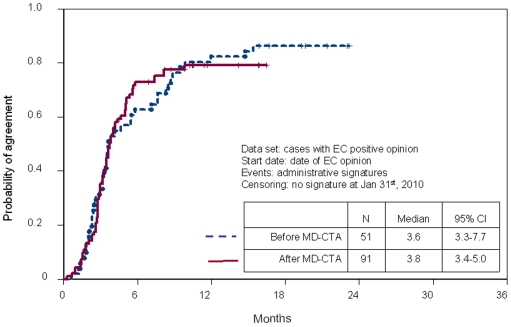
Time to signature of administrative agreement after a positive EC opinion. Vertical lines represent censored applications.

After a positive EC opinion (**figure 4**), there was no difference before and after MD-CTA in the time required to sign the administrative agreements (median 3.6 and 3.8 months, respectively).

**Figure 5:**
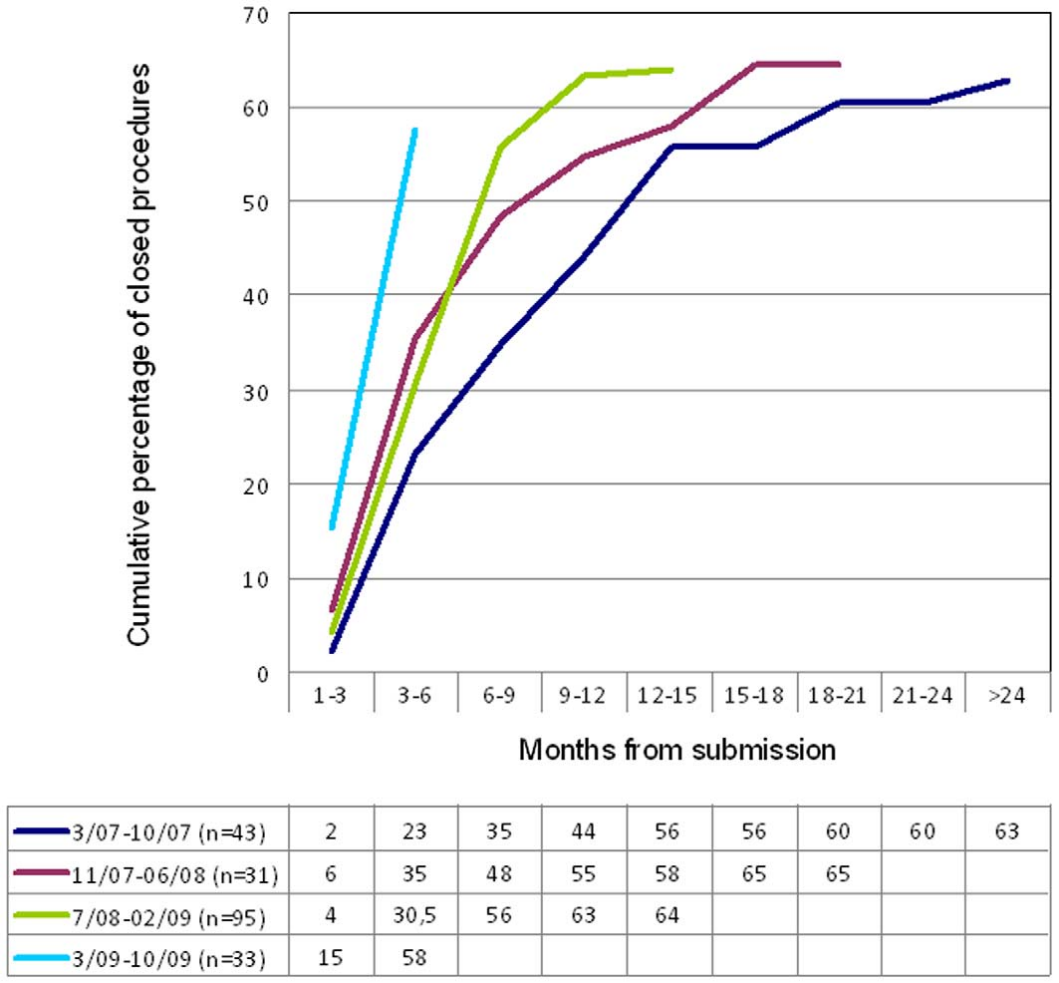
Cumulative percentage of applications, divided in four time cohorts, coming to closure over the time.

A trend toward shortening time to closure of the whole submission process over time is evident in the graphical description reported in **figure 5**. Indeed, the rate of submissions that reached closure (either signature of the agreement or a negative EC opinion) within 6 months from the date of submission has increased from 23% in the oldest cohort to 58% in the most recent one; however, in the latter cohort, only 15% of the submission came to closure within 3 months.

## Discussion

Our study shows that there is an encouraging trend toward shortening the time spent for activation of non-profit studies in oncology in Italy, in the experience of a single promoting centre. Such shortening does not seem related to changes in the time required for administrative agreement signature that did not vary before and after MD-CTA. Rather, it seems that it may derive from a higher efficiency of EC. However, we cannot draw definitive conclusions on the role played by the MD-CTA because we see shorter time to EC opinion after MD-CTA only in the more biased comparison that include EC involved only before or only after MD-CTA; therefore, it is reasonable that the observed difference is due to a selection bias of EC, rather than to the effect of MD-CTA.

However, the whole process remains inefficient, with about a 40% failure rate, quite high considering that all submissions in our case were prompted by the declared interest of investigators at participating centres. In addition, it is regrettable that, in the most recent and best performing cohort of submissions, only 15% of procedures came to closure within 3 months. Such lack of efficiency is extremely burdensome for promoters, particularly the non-profit ones always working with limited economic resources.

Application failures are primarily due to lack of EC response and administrative reasons, and only rarely to a negative opinion and a refusal by EC. We believe that the high number of EC plays a role in the lack of efficiency. According to the last version of Bulletin of the National Monitoring Centre for Clinical Trials, 269 EC are active in Italy [10]. It is reasonable that, within such a high number of EC, many lack minimum structural requirements to work efficiently; these committees actually prevent the possibility of clinical investigators and their patients to participate in clinical trials that might in some cases offer positive chances of treatment.

The administrative approval process seems completely unaffected by MD-CTA, in our experience, although the EU directive was intended to harmonize administrative procedures as well as the work of EC. However, the directive is not mandatory regarding time allowed for administrative approval, further, administrative authorities might lack sensitivity to the value of clinical trials and their management. This is clearly supported by the fact that, in our experience, administrative failure thwarted a positive EC opinion in 27 of 142 cases. This is hard to accept, even if this figure might be overestimated because some of the most recent applications could become successful after the limiting date chosen for the analysis.

Our data are consistent with literature suggesting that steps and time required to develop and activate multicentre clinical trials are too extensive and the development time is overly long and highly variable [11], [12]. In particular, prolonged delays in obtaining regulatory approvals to initiate clinical trials is reported in many countries, where obtaining approval can take as long as 6 to 9 months [13], a time so long to be unethical [14].

Therefore, major efforts should be made to reduce and standardize procedures concerning the approval of clinical trials, also focusing on administrative procedures, because time to trial activation is critical for clinical research.
